# Effect of Charging Parameter on Fruit Battery-Based Oil Palm Maturity Sensor

**DOI:** 10.3390/mi11090806

**Published:** 2020-08-25

**Authors:** Norhisam Misron, Nisa Syakirah Kamal Azhar, Mohd Nizar Hamidon, Ishak Aris, Kunihisa Tashiro, Hirokazu Nagata

**Affiliations:** 1 Faculty of Engineering, Universiti Putra Malaysia, Serdang, Selangor 43400, Malaysia; mnh@upm.edu.my (M.N.H.); ishak_ar@upm.edu.my (I.A.); 2 Institute of Advance Technology (ITMA) and Institute of Plantation Studies, Universiti Putra Malaysia, Serdang, Selangor 43400, Malaysia; 3 Faculty of Engineering, Shinshu University, Wakasato 4-17-1, Nagano 380-8553, Japan; tashiro@shinshu-u.ac.jp; 4 Centre for Global Education & Collaboration, Shinshu University, Wakasato 4-17-1, Nagano 380-8553, Japan; hnagata@shinshu-u.ac.jp

**Keywords:** oil palm maturity sensor, fruit battery base, load resistance, charging voltage, charging time

## Abstract

Oil palm is one of the key industries highly observed in Malaysia, due to its high demand both whether locally or internationally. The oil extraction rate (OER) in palm oil production is used as an element to identify the performance of the mills, estates and producers. In view of this, there are specific instrument or sensor needs to be implemented at the mills especially during the reception of fresh fruit bunches (FFB) transported from the field for oil content processing. This paper aims to study and propose the use of a fruit battery-based oil palm maturity sensor to analyse the effect of the sensor to various parameters. The study utilizes a charging method with different parameters, including a moisture content test on the palm oil samples. Three types of parameters are tested along with the different grades of oil palm fruit from different bunches, such as the load resistance, charging voltage and charging time. The repeatability data of the samples are obtained with the used list of values in each parameter. The results show that the parameters tested for the unripe, under ripe and ripe samples can affect the sensor sensitivity.

## 1. Introduction

The oil palm production plays a major role in Malaysia’s economy, due to high global demands. Starting from only one main destination for exportation, now the products of Malaysia’s oil palm is exported to 193 markets worldwide. The growth and success of the oil palm industry in Malaysia is making it one of the great contributors in Malaysia’s Gross Domestic Product (GDP) [1]. Apart from that, Malaysia is considered to be the second largest exporter and producer of crude palm oil (CPO) [2]. However, there are fluctuations of the CPO production in certain years, due to several factors, for example, due to the low quality processing of palm oil from the fresh fruit bunches (FFB).

It is known that it takes around six months for the FFB to mature before being harvested [2]. Moreover, the fresh fruit bunch per hectare can be indicated as the yield of the palm oil output production, while the oil production from the FFBs signifies the oil extraction rate (OER). This shows that the OER is one of the tools to measure the estates’ or mills’ performances [3]. This is important when considering what happened to Felda Global Ventured (FGV) before 2017, due to unripe bunches being sent from the settlers, which could not be processed, and this caused them to loss 80 million Malaysian Ringgit (MYR) [4]. Hence, the estates and mills play a major role in ensuring that the harvesting of the field be good in every aspect to produce great quality OER.

The oil palm mill process comprises specific procedures and policies, starting from the harvesting of the FFBs to the production of the palm oil. The harvesters tend to cut the ripe together with the unripe bunches during the low crops period, in order to retain earnings, which then affects the quality of the palm oil production. In addition, some of the ripe bunches left out during the high crop period become overripe by the next harvesting round. Furthermore, some FFBs that are forgotten or left out for transportation also become overripe or not fresh anymore by the next harvesting round [5]. The FFB then transported to the mills has to go through a screening process first before undergoing the further OER process. According to the Malaysian Palm Oil Board (MPOB) standard, the visual inspection by a human is one of standard procedures used to grade the oil palm fruit. This is one of the factors that contributes to low OER production, which is why the separation of the FFB at the mills after the reception process plays an important role to determine the ripeness of the FFB. This is to ensure that the FFB chosen has a high oil content and is ripe enough to be processed. Thus, various technologies and methods have shown the importance of oil palm grading.

Various methods are introduced to identify the oil palm fruit maturity grading with different detection methods. The most popular method is to use imaging techniques and computer vision software to identify the oil palm grading [6,7,8,9,10,11,12]. Other techniques are to identify the oil palm ripeness by using colour vision or red, green and blue (RGB) to evaluate the FFB grading [13,14,15]. You et al. [16] introduced a microwave technique to monitor the oil palm ripeness in Malaysia. Another study of oil palm ripeness determination uses a hyperspectral device and machine learning algorithm by Bensaeed et al. [17]. The different wavelengths from different types of oil palm grading are classified by an artificial neural network. Moreover, a study using the neural network method to identify the ripeness of the oil palm is proposed by Ibrahim et al. [18]. Zolfagharnassab et al. [19] proposed research to identify the oil palm bunch ripeness by its mean temperature by implementing a thermal sensor prototype. A novel inductive concept frequency technique is proposed by Harun et al. [20] to investigate the grading of oil palm bunches. The inductive concept frequency technique then is improved for its sensitivity [21]. Harun et al. [22] then developed the dual flat-type shape of the air coil sensor. Further experiments on moisture or water content determination in the oil palm samples was studied in the inductive type of research [23]. The inductive sensor of dual flat-type is further studied and developed as the triple flat-type by Aliteh et al. [24], with a moisture content experiment that is the same as that of the authors of Reference [23]. In addition, Junaidah et al. [25] proposed a sterilization process at a certain temperature and time in order to determine the oil palm ripeness. Thus, various technologies and methods have been invented to measure the oil palm fruit bunch ripeness and shows the importance of identifying the oil palm ripeness.

This paper aims to further study the enhancement of the fruit battery-based oil palm maturity sensor according to the concept as introduced from previous experimental research [26,27,28], in which the development of the maturity sensor was proposed in this recent research paper [29]. Previously, fruit battery research generally uses zinc and copper as the electrodes, but in this research, aluminium is used to replace the copper. In this paper, a fruit battery with a charging concept is developed on the basis of three different parameters which are tested in this experimental research. These are: load resistance, charging voltage and charging time. The objectives of this invention are to determine the ripeness of different types of oil palm fruits and to obtain consistent results from the sensor measurement. Moreover, the effect of the parameters on different oil palm grades is also one of the objectives studied in this paper. The moisture content determination is applied throughout this research in order to analyse the effect of this fruit battery sensor along with the moisture content.

## 2. Fruit Battery Base Oil Palm Maturity Sensor

### 2.1. Sensor Application

**Figure 1:**
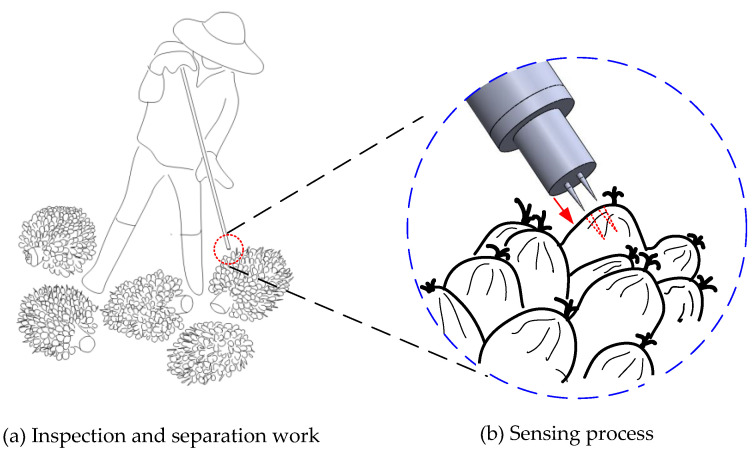
FFB (fresh fruit bunches) inspection using a fruit battery sensor.

The processing of oil palm FFB requires several procedures with a specific policy, starting from the harvesting of the FFB and the transportation of the FFB to the mills to the processing of the FFB to become palm oil: crude palm oil and palm kernel oil. All of the steps included require high monitoring aspects to deliver a quality target of oil production on the basis of the mills’ performance. It is known that oil palm FFB usually takes about 18 weeks or more for harvesters to cut and collect. The FFBs that are cut are transported from the oil palm field to the mills to be processed. The palm oil is produced from several processes at the mills such as inspection, sterilising, stripping and other further processes. This paper focuses on using the sensor during the inspection of the FFB at the mills’ reception, as illustrated in Figure 1.

According to the MPOB manual, the colour of the ripe fruit is reddish-orange with 10 or more empty sockets on the bunch. However, it is not possible to detect the actual oil content of the FFB using the manual inspection with the naked eyes. This method of inspection might not be accurate especially if the FFBs sent to the mills are not of the correct ripeness level, which leads to a lower oil content extraction. Nowadays, colour vision or computer vision is being implemented at the mills to distinguish the ripe bunches. However, this method requires a lot of steps or procedures which take longer for the worker to separate the bunches. Thus, the fruit battery sensor with a charging concept is proposed in this paper to be used as a sensor to detect the ripeness of the oil palm fruit during FFB inspection. The results obtained throughout the research by obtaining a load voltage *V*_L_, and moisture content *w* determination can be used as a reference to distinguish the bunches that have more oil content.

### 2.2. Basic Principle Operation

**Figure 2:**
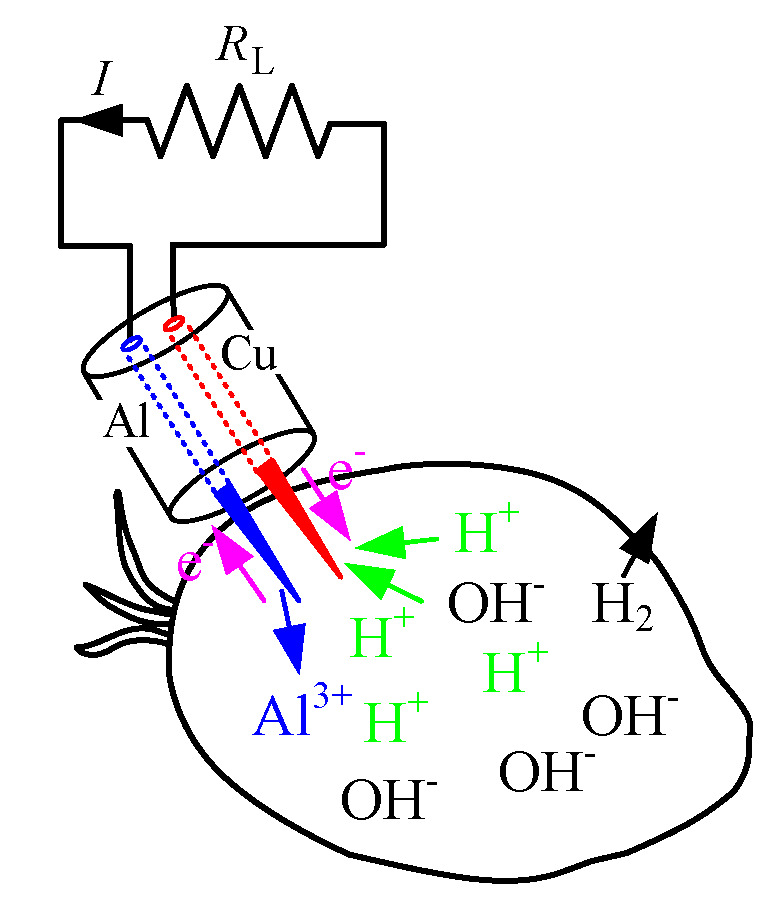
Fruit battery sensor concept.

On the basis of the previous research [29], this study uses the same electrochemical cell to determine the ripeness of the oil palm fruits. Aluminium (Al) and copper (Cu) material are still used as an electrochemical cell sensor by using the fruit battery with a charging concept. The sensor is tested on different types of oil palm grading from different trees of FFB: unripe, under ripe and ripe. The chemical reaction of the fruit battery using aluminium and copper electrodes are illustrated in Figure 2. In this experiment, the oil palm fruit content acts as an electrolyte to the reaction. The aluminium and copper electrodes are then pricked into the fruits, the aluminium ions (Al^3+^) are charged as the atom of aluminium is dissolved in the electrolyte, after which the three negatively charged electrons (e^−^) are left behind. Three positively charged hydrogen ions (H^+^) from the electrolyte combine with three electrons at the surface of the copper electrode, by which the hydrogen molecule (H_2_) is formed. The electrons used to form the hydrogen molecule are then moved from the aluminium to the copper through the external wire so that the electric current is generated.

The chemical reaction of the fruit battery is shown below [30].

Al (s) → Al^3+^ (aq) + 3 e^−^ (1)

(2) $$3\;H^{+}(\mathrm{aq})\;+\;3\;e^{-}\;\to \;\frac{3}{2}\;H_{2}\;(g)$$

### 2.3. Charging Concept

The fruit battery sensor is applied with the charging voltage by using an electrical insulation-continuity tester Model 3132A by Kyoritsu. Before the charging concept is applied, the load voltage *V*_L_ generated is not stable, which might cause the inaccuracy of the result. The load resistance voltage obtained from the oil palm fruit is generated in mV which shows the sensitivity of the sensor. Thus, the charging concept is applied to the circuit for a certain period before the fruit battery experiment is connected.

**Figure 3:**
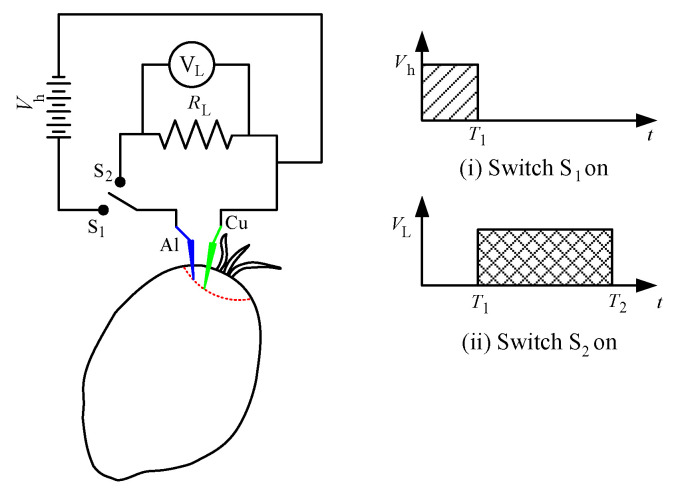
Charging concept with a switching mechanism.

The purpose of applying the charging voltage to the fruit battery sensor is to obtain the steady-state condition of the reading for different types of oil palm grades. From Figure 3, the schematic diagram of the experiment with charging concept is shown together with the switching operation for further explanation. As the power supply *V*_h_ is turned on while switch S_1_ is connected, the voltage is supplied to the circuit and the fruit is charged. While the voltage injection is applied onto the fruit, the fruit battery circuit is not connected at this period. This part is called the charging concept applied to the experimental research. After a certain period, which is called charging time *t*_c_, switch S_2_ is connected immediately, after which the fruit battery reaction occurs. The chemical energy of the fruit battery is converted to electrical energy as the load voltage *V*_L_ is generated. This stage, in which the load voltage is generated after the fruit is charged, is called a fruit battery experiment. The readings obtained from the experiment show the characteristics of the chemical energy discharged from the oil palm fruit, and the load voltage value shows an amplified value as the fruit battery circuit is turned on due to the electrical energy supplied to the fruit. Furthermore, the switching part between S_1_ and S_2_ happens in an instantaneous time to obtain the *V*_L_ when the energy is released from the fruit at a particular time. Hence, in one experimental setup, there are two parts of the concept applied which are the charging concept and fruit battery experiment. The experimental setup is based on the figure below for a further test implementation.

## 3. Experiment Parameters and Evaluation Method

### 3.1. Parameters Selection

The fruit battery research has been studied and developed with different materials used as the electrodes such as magnesium and zinc. All those materials used however have flexible material which are not appropriate for testing on the hard skin of oil palm fruit (unripe). In this study, aluminium (Al) and copper (Cu) are chosen as the electrode’s material because of the availability and material strength. The aluminium and copper material used is fabricated with a pin shape for its sensor head. The sharp tip of the electrode allows the sensor to be pricked onto the fruit surface, due to the large total surface area. Oil palm grading comprises different grading such as ripe, under ripe and ripe. This is especially true, since the under ripe and unripe type of FFBs have hard flesh. Therefore, the shape of the electrodes used in this research is the first important aspect, so that the sensor can be pricked to any type of FFB easily.

This paper focuses on the three different parameters of the charging concept applied to the fruit battery sensor: load resistance, charging voltage and charging time. Load resistance *R*_L_ is used in this experiment to determine the load voltage drop as the fruit battery switch is connected. Since the charging method is implemented in the fruit battery sensor, different values of voltage injection are used to study their effect of the sensor performance. These are: 250 V, 500 V and 1000 V. This is called the charging voltage, in which the voltage is injected into the sample when switch S_1_ is turned on. Furthermore, charging time *t*_c_ is the timing of the voltage injection supplied to the oil palm fruit before the experiment is switched to the fruit battery experiment (switch S_2_). This is implemented to analyse the sensor sensitivity when the fruit is charged in different times. These times are 5 s, 10 s and 15 s. The parameters are tested repeatedly (3 times) in the samples with different values set throughout the experiment to obtain a certain trend for the consistency of results. The three parameters comprised of load resistance, charging voltage and charging time values are listed in Table 1.

### 3.2. Experimental Setup

**Figure 4:**
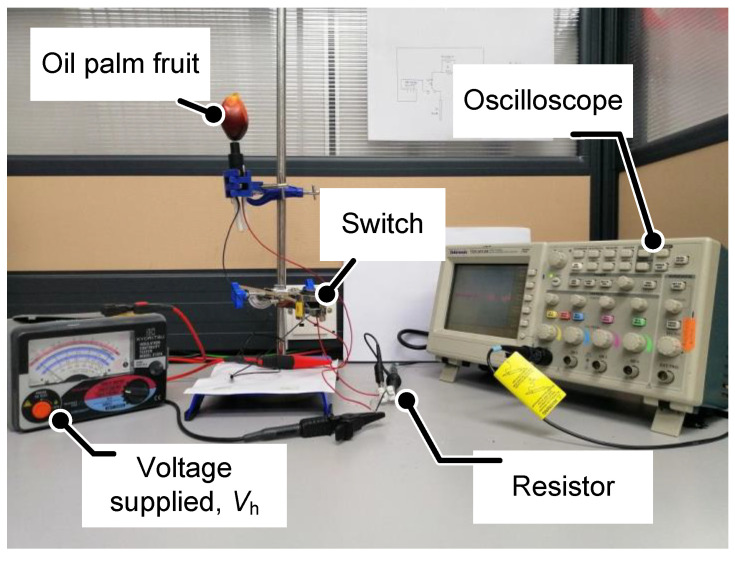
Experimental setup of fruit battery sensor with charging method.

Figure 4 below shows the experimental setup of the fruit battery experiment with the charging concept method. The fruit is charged from the supply *V*_h_ as switch S_1_ is turned on before switching to fruit battery circuit (switch S_2_). Every single sample is tested with different specification values by implementing every possible outcome. Based on the figure, the result of the voltage drop which happened at the resistor is displayed and recorded through the oscilloscope in real-time reading. The data is taken three times to obtain a convincing load voltage *V*_L_ result. In this experiment, the range of fruit tested is around 20 to 30 samples per every different experiment parameter. The proper steps for handling this experiment is taken into consideration, as the sensor is very sensitive. The proper sanitization of the sensor electrodes is conducted by cleaning the surface of the aluminium and copper electrodes repeatedly each time before the next set of experiments is conducted. This is done to avoid the contamination of any foreign particles on the electrodes that could affect the results. Besides, the specific software application of OriginLab is used to generate and analyse the data obtained from the digital oscilloscope.

### 3.3. Moisture Content

**Figure 5:**
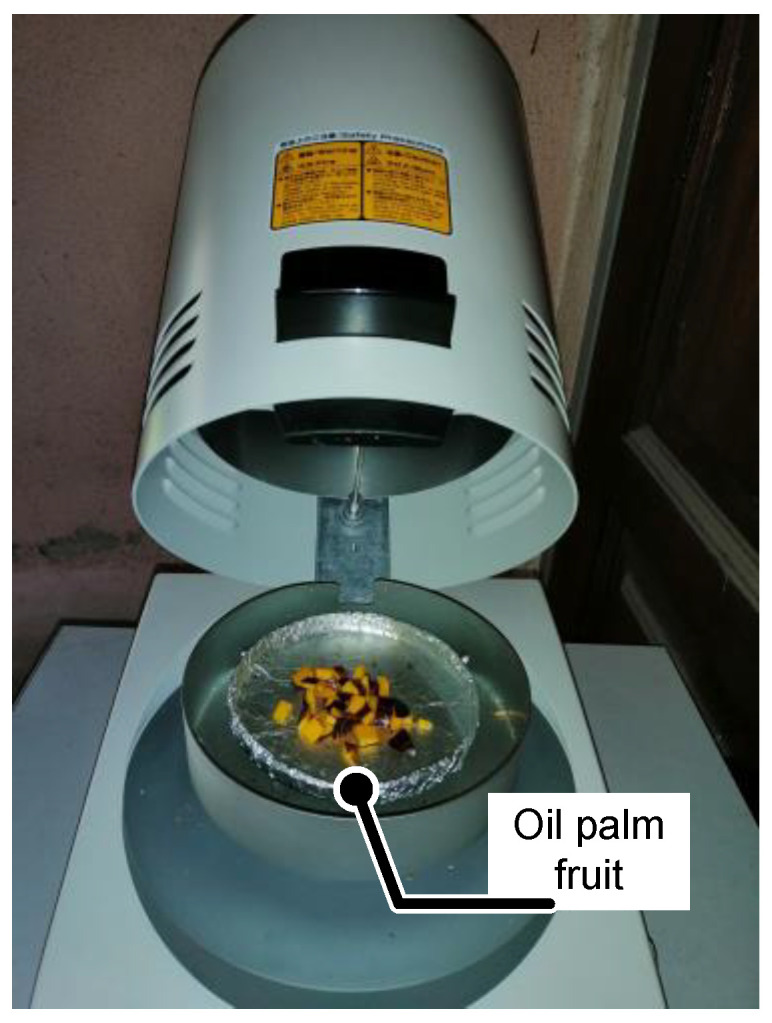
Drying process for moisture content.

The samples tested are taken for the moisture content determination on the same day. Each fruit tested is submitted for the drying process after the fruit battery experiment is completed. Around 75% of the oil palm fruit flesh is cut into small pieces before weighing and drying. Moisture content determination is done for each of the samples, with a temperature of 115 °C for 60 min, by using an infrared moisture determination balance FD-610 by Kett Electric Laboratory, as shown in Figure 5. The samples are cut into small pieces and dried at the desired temperature. The oil palm that is cut into small pieces has a larger total surface area of the fruit flesh, which yields a higher accuracy of the moisture content. The weight of the sample before and after the drying process is taken and calculated with the formula below.

(3) $$\mathrm{Moisture}\;\mathrm{content},\;w\;\left(\%\right)=\frac{\mathrm{Weight}\;\mathrm{before}}{\mathrm{Weight}\;\mathrm{after}}\times 100$$

### 3.4. Data Evaluation

**Figure 6:**
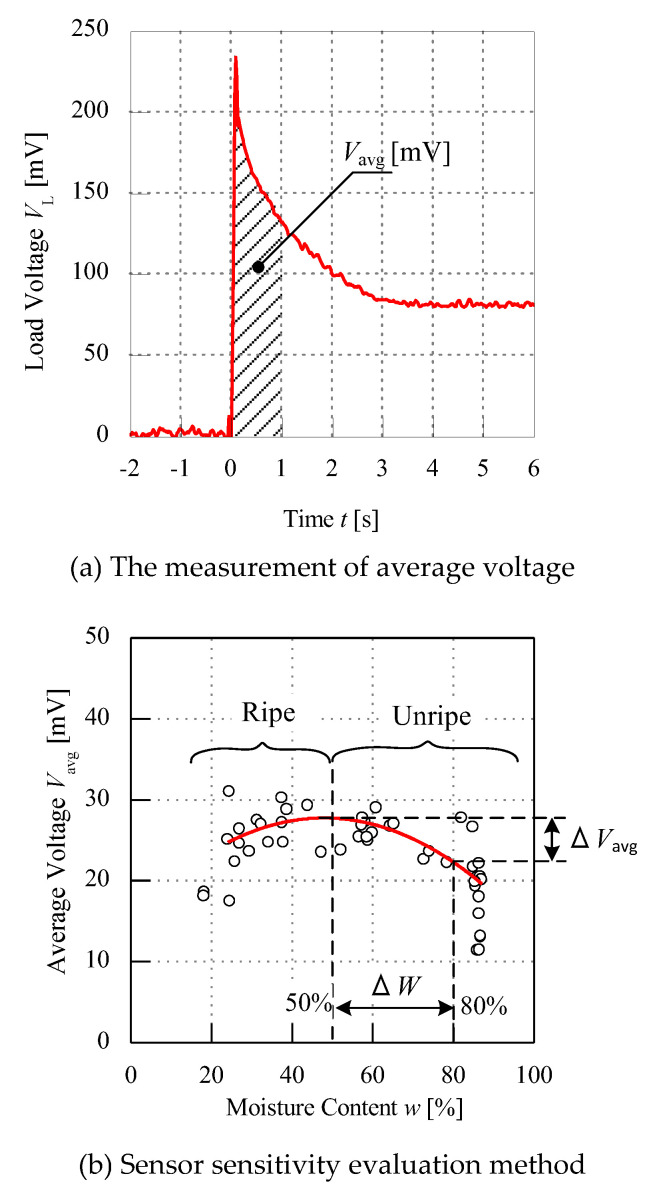
Data evaluation.

The data is comprised of the load voltage *V*_L_ and moisture content *w* of all the samples tested. Figure 6 shows the evaluation of the raw data of the sample obtained from the oscilloscope reading. OriginLab software (OriginPro 2015, OriginLab Corporation, Northampton, MA, USA) is used to identify and generate simple mathematical expressions for data extraction. Figure 6a shows the raw data of one condition of a sample after the noise has been filtered. Since the raw data of the fruit battery experiment points out that each load voltage versus time has a volatile characteristic, the filter method is applied to ensure there is no noise in the graph or other rapid scale. As seen in Figure 6a, the red line is the raw data obtained directly from the oscilloscope reading after been filtered. To obtain the average voltage *V*_avg_, one second from the peak load voltage *V*_L_, as shown in the shaded area, is analysed and simulated to get its area under the graph. This shaded area unit is in mVs, and after it is divided by one second to obtain the average for one test, it is in mV. This data extraction and simulation shown is applied to one single sample at one condition. Hence, the test is repeated three times to one single sample and all of the average voltages of three tests is averaged again to obtain one point of average data before plotting it together with the moisture content.

The *V*_avg_ from all the samples with different conditions is then plotted as illustrated in Figure 6b. The *y*-axis of the graph is the average voltage *V*_avg_ calculated from the software while the *x*-axis is the moisture content *w* of the samples from the drying process. From the graph, there are two types of oil palm fruit maturity that can be interpreted according to the ripeness, which are ripe and unripe. There is a difference of the ripe and unripe fruit based on its average voltage *V*_avg_. The average voltage *V*_avg_ following moisture content values can be seen from ripe to unripe. The average voltage *V*_avg_ shown is dependent on the sensitivity of the sensor performance. The sensitivity of the sensor is illustrated on the ratio of average voltage *V*_avg_ of the samples with relation to moisture content *w*, which will be explained in the results. The polynomial curve fitting line (red line) is generated in the graph to interpret the visualization of the data points obtained. Besides, the analysis based on the moisture content can show the difference of the voltage average ∆*V*_avg_ of ripe and unripe samples. Ripe samples are considered below moisture content *w* 50% because ripe fruits can appear as reddish-orange according to the MPOB standard, which then can be identified by the work at the mills through visual inspection. The moisture content difference ∆*W* of unripe fruit is observed between 50% to 80% of moisture content *w* and the average voltage *V*_avg_ and can be classified between the region 50% < x < 80%. Thus, in this paper, only the unripe part of the graph of voltage difference ∆*V*_avg_ is analysed and explained.

**Figure 7:**
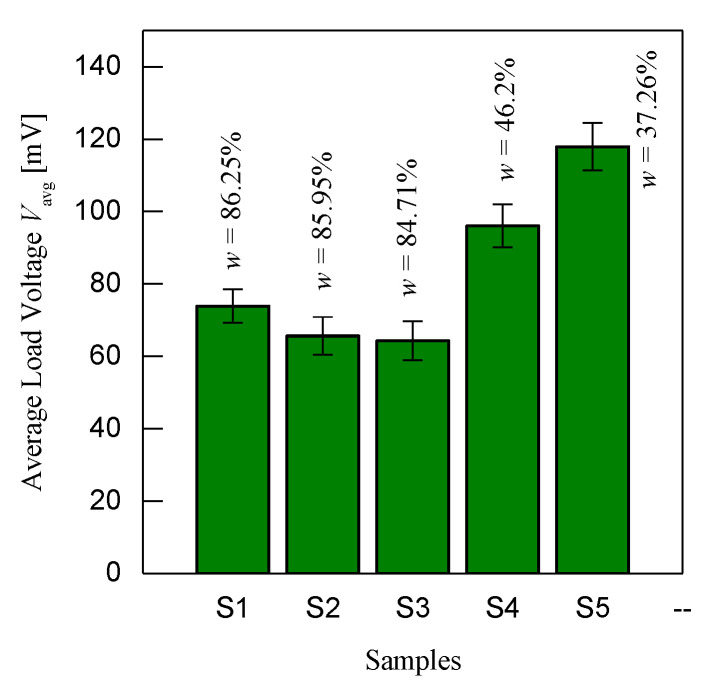
Example of the mean with standard deviation (Y-error) bar chart when *R*_L_ = 500 Ω, *t*_c_ = 5 s and *V*_c_ = 250 V.

Figure 7 is shown below to show an example of the data analysis with a further explanation in Figure 6a. The bar chart shown is one of the examples of several samples but tested with one condition parameter *R*_L_ = 500 Ω, *t*_c_ = 5 s and *V*_c_ = 250 V. As seen in the bar chart, sample 1 is tested three times with one condition and this method is applied to other samples as well. The *x*-axis is the average load voltage *V*_avg_ and the *y*-axis is the sample of the data that has been averaged. This average value of one sample is the point that makes up the one-point data, as shown in Figure 6b. From the bar chart, the Y-error of all samples is calculated and indicated in the figure. The Y-error can be seen to have similar representations to each other, which shows the stability of the sensor to determine the ripeness of the oil palms that are stable, thereby assuring to obtain less error in the data.

## 4. Sensor Characteristics and Performances

### 4.1. Sensor Performance Due to Different Charging Voltage

**Figure 8:**
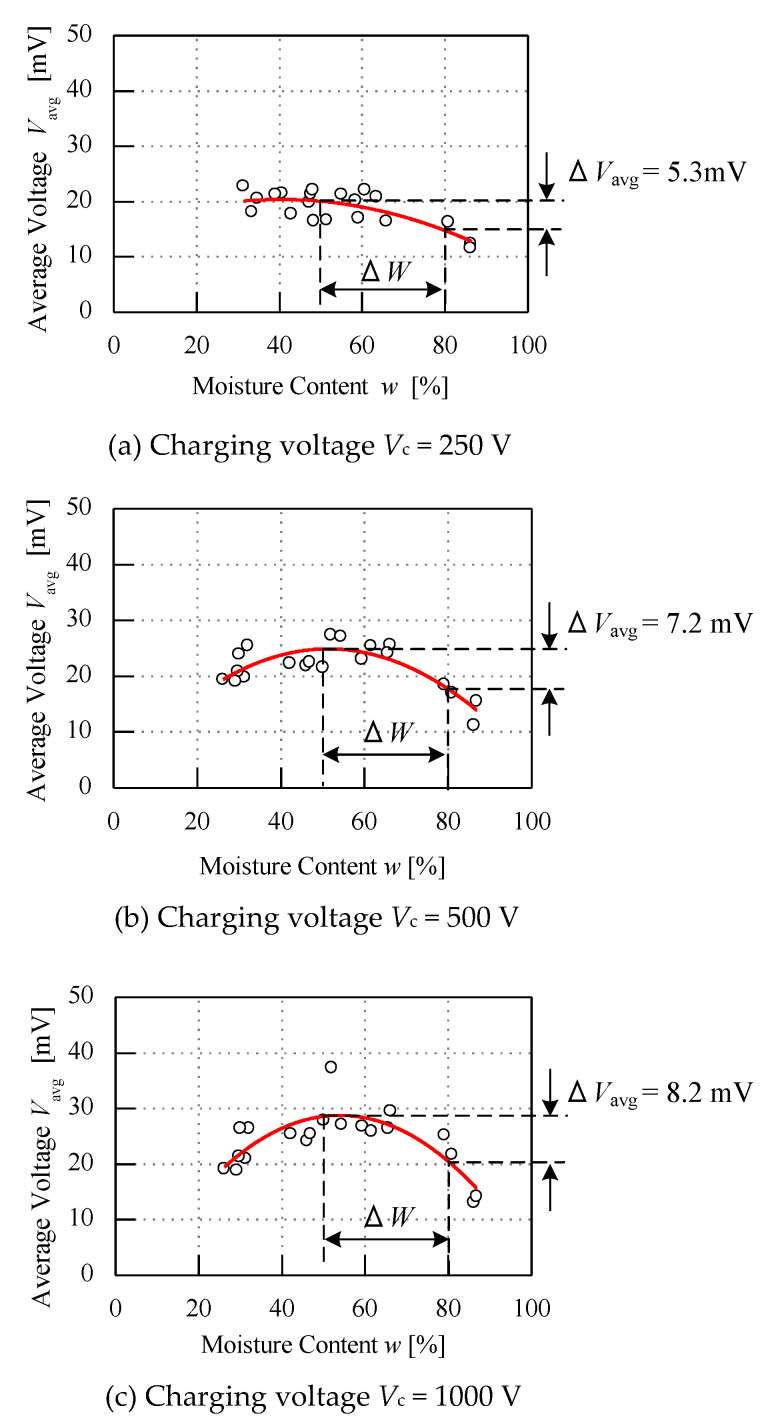
Graph of different voltage injection.

Several parameters are tested to study the performance of the sensor. This part is further explained to study the effect of the sensor performance of various charging voltages with a constant load resistance *R*_L_ and charging time *t*_c_. Figure 8 below shows three graphs when the different charging voltage is supplied. In this condition, the constant of load resistance *R*_L_ = 100 Ω and charging time *t*_c_ = 5 s are used with various charging voltage *V*_c_ values. Based on the graphs below, the average voltage *V*_avg_, when the different charging voltage is applied, shows a different curve fitting to each other. As seen from the *y*-axis of the average voltage *V*_avg_ on all graphs, as the charging voltage increases, the average voltage *V*_avg_ of the samples increases. The polynomial fit line that can be seen lies higher, from 250 V to 1000 V, and is curvier. When comparing the ∆*W* range from 50% to 80%, the difference voltage ∆*V*_avg_ increases when the charging voltage *V*_c_ increases. As seen from the graph, when the charging voltage *V*_c_ is supplied with 250 V, 500 V and 1000 V, the voltage difference ∆*V*_avg_ increases to 5.3 mV, 7.2 mV and 8.2 mV, respectively. The sensitivity of the sensor increases by 36% when the charging voltage *V*_c_ is increased from 250 V to 500 V. Eventually, the sensitivity of the sensor performance increases by about 55% from 250 V to 1000 V. Hence, the effect of increasing the charging voltage *V*_c_ increases the average voltage *V*_avg_ and voltage difference ∆*V*_avg_.

### 4.2. Sensor Performance Due to Different Charging Time

**Figure 9:**
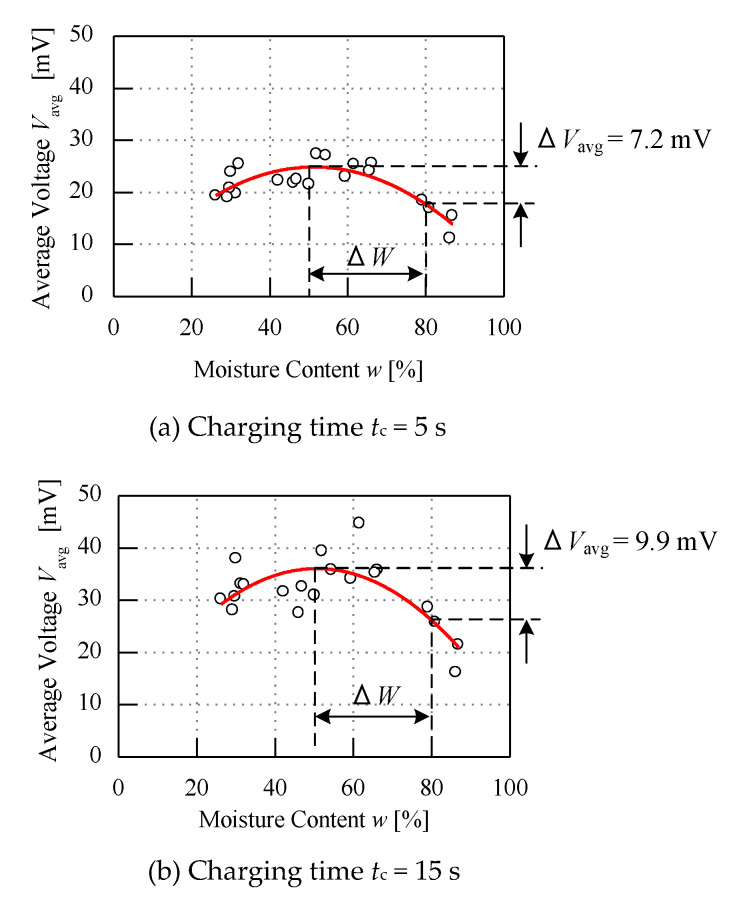
Graph of different charging time.

The experiment is continued by testing with different conditions of parameter specifications, and in this part values of *R*_L_ = 100 Ω and *V*_c_ = 500 V are kept constant while the *t*_c_ is varied, as shown in Figure 9. Based on the graphs, the curve or line of the graph clearly shows the trend of the ripe and unripe average voltage *V*_avg_ obtained. On the basis of the range of unripe fruit moisture content from 50% to 80%, it can be seen that the line of the curve is aligned around 10 mV to 30 mV when the charging time is set to 5 s, while for charging time *t*_c_ at 15 s, the curved line for the unripe part is aligned after 10 mV until it is near to 50 mV. The ∆*V*_avg_ increases when the charging time *t*_c_ is increased. From the figure, when *t*_c_ is tested for 5 s, the difference of the average load voltage ∆*V*_avg_ is 7.2 mV, while for 15 s of charging time *t*_c_, the voltage difference ∆*V*_avg_ is 9.9 mV. Around 38% of the sensitivity of the sensor increases when the charging time *t*_c_ is tested from 5 s to 15 s. Thus, the longer the charging time *t*_c_, the higher the increase of the average voltage *V*_avg_ and the voltage difference ∆*V*_avg_. This is because when the charging time *t*_c_ is longer, the fruit receives more energy, which will then cause the high spike in the voltage of the raw data when switch S_2_ is connected.

### 4.3. Sensor Performance Due to Different Load Resistance

**Figure 10:**
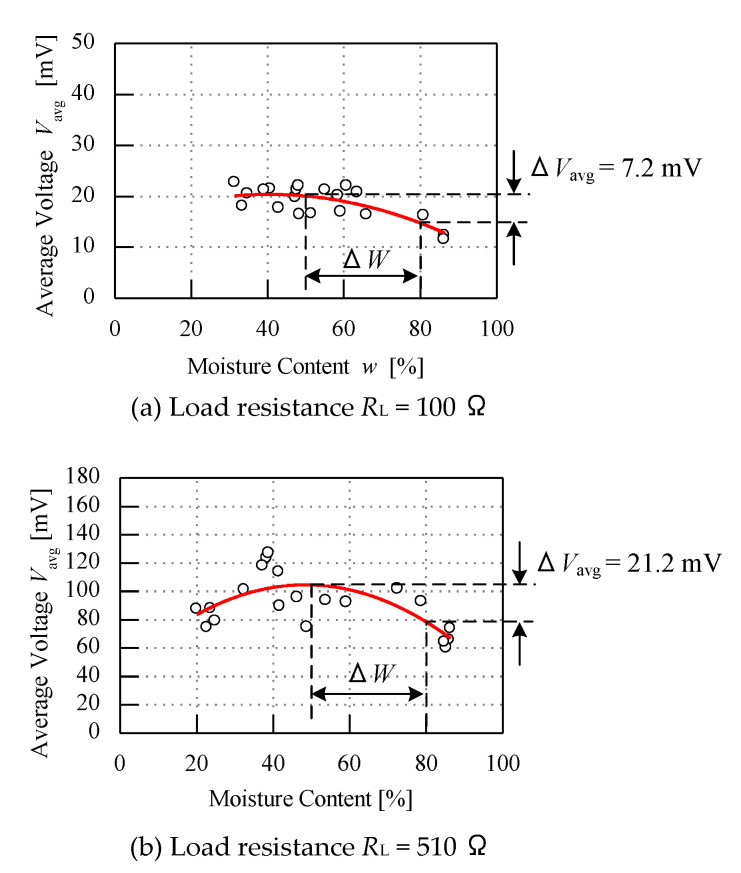
Graph of different load resistance.

The experiment of the fruit battery sensor is further tested with different *R*_L_ values while the value of *t*_c_ and *V*_c_ are kept constant at 5 s and 250 V. In this part of the changing load resistor, the graphs show a significant increment of the average voltage *V*_avg_. On the basis Figure 10, two values of load resistor are used, which are 100 Ω and 510 Ω, respectively. The curved polynomial line when using *R*_L_ = 100 Ω is aligned between 10 mV and 30 mV. Besides, when the load resistor *R*_L_ is changed to 510 Ω, the curved line could be seen to be aligned between 60 mV and 140 mV. From the graphs, when the load resistance *R*_L_ is tested with 100 Ω and 510 Ω, the voltage difference ∆*V*_avg_ obtained is 7.2 mV and 21.2 mV, respectively. The results have seen the sensitivity increase by three-fold of its sensitivity. Thus, increasing the load resistance *R*_L_ will increase the average voltage *V*_avg_. This shows that the higher resistance value will produce the steady-state condition of the data obtained compared to the lower resistance value.

### 4.4. Sensor Performance

**Figure 11:**
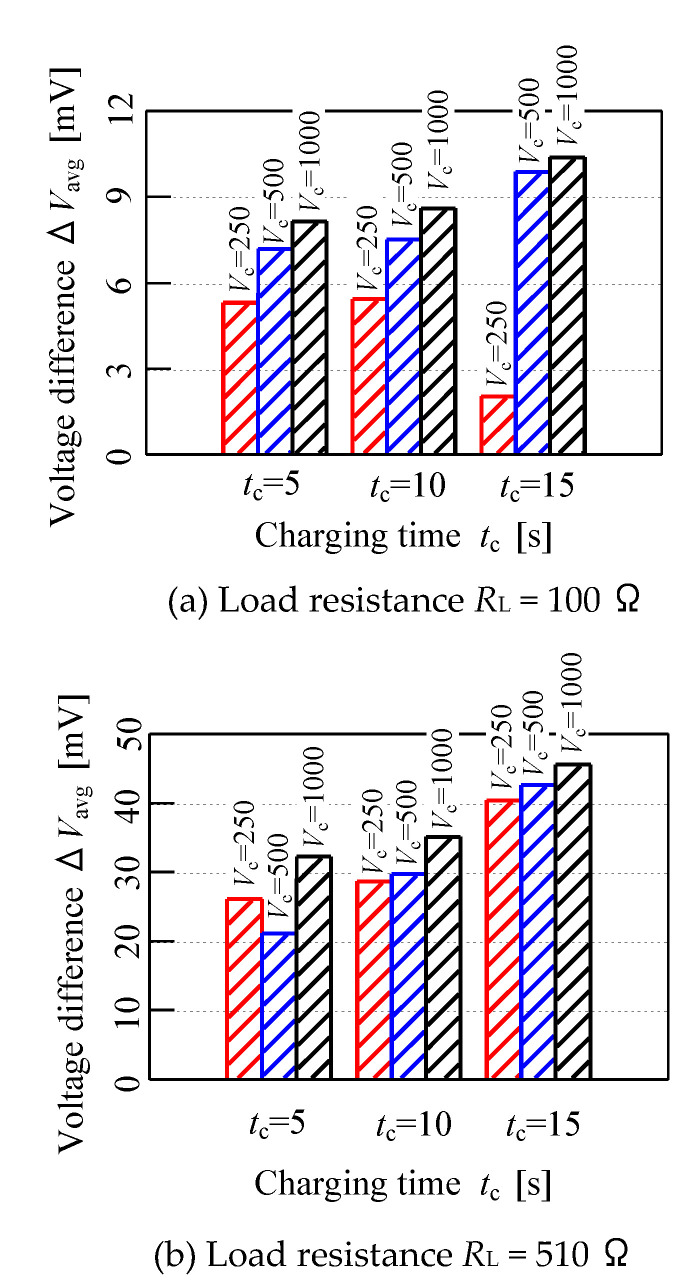
Bar graph of voltage difference for different sensor condition.

The performance of the sensor when tested with different conditions of parameters specification is illustrated in Figure 11. In this experiment, the effect of the sensor performance could be seen when the parameters of specifications were tested with specified values. Figure 11 below is divided into two parts to illustrate the voltage difference ∆*V*_avg_ in the overall conditions when tested with the fruit battery sensor. Generally, the voltage difference ∆*V*_avg_ increased when the charging time *t*_c_ and charging voltage *V*_c_ increased, as shown in the load resistance *R*_L_ of 100 Ω and 510 Ω graph. Moreover, the data shows that the sensor performance increases stably from the analysis between 50% and 80% of moisture content ∆*W*. When comparing the red bar in both graphs, the voltage difference ∆*V*_avg_ increases when the charging time *t*_c_ and load resistance *t*_c_ is increased. Moreover, the blue and black bar graph of load resistance *R*_L_ = 100 Ω shows that the sensor performance increases in its voltage difference ∆*V*_avg_ when the *t*_c_ and *V*_c_ increase and the same also goes for the blue and black bar in 510 Ω graph. Furthermore, by observing the *y*-axis of ∆*V*_avg_, the unit mV triggered when using load resistance *R*_L_ = 510 Ω is higher compared to 100 Ω. Thus, the sensitivity of the sensor performance can be identified from the bar graph shown below.

### 4.5. Sensor Distribution Chart

**Figure 12:**
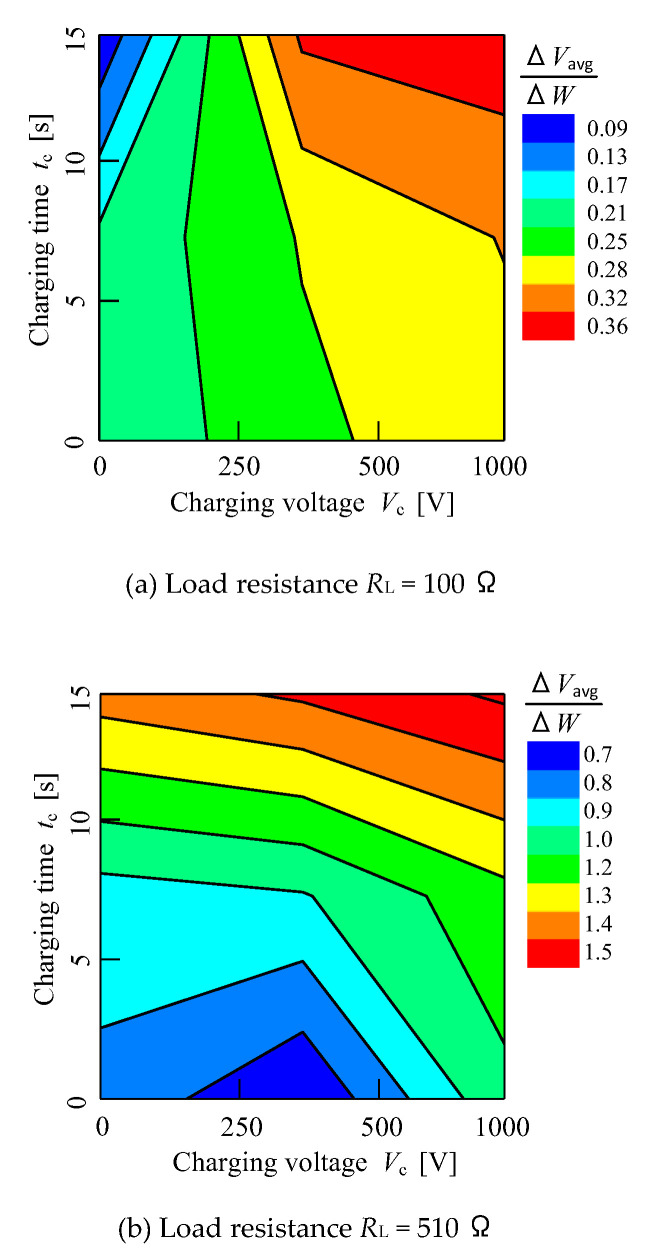
Distribution chart of average load voltage to moisture content ratio.

The voltage difference ∆*V*_avg_ data can be identified by its ratio with moisture content ∆*W* of all the samples tested. Figure 12 shows the distribution chart of charging time *t*_c_ against charging voltage *V*_c_. The colour grading shown on the right side of the graph is the ratio of voltage difference ∆*V*_avg_ and moisture difference ∆*W* which is $\frac{∆V_{\mathrm{avg}}}{∆W}$. In this part, the sensitivity determination of the sensor is identified from the slope of the graph which is the ratio of voltage difference ∆*V*_avg_ and moisture difference ∆*W*. This distribution chart is shown to explain the sensitivity of the fruit battery sensor between 100 Ω and 510 Ω of load resistance *R*_L_ that is used in the experimental research. The sensitivity of the sensor could be interpreted by referring to this colour region. From the figure, when the charging voltage *V*_c_ and charging time *t*_c_ are higher, the colour will eventually lie on the red colour. For example, when the charging voltage *V*_c_ is supplied at 1000 V within 15 s charging time *t*_c_, the ratio falls on the red colour region. Another condition example is the following: when 500 V of charging voltage *V*_c_ is tested within 10 s, the ratio value is 0.32 (orange colour) in load resistance *R*_L_ = 100 Ω and 1.3 (yellow colour) in the 510 Ω chart. Moreover, the sensitivity of the sensor can be analysed from the ratio of voltage difference ∆*V*_avg_ to moisture content difference ∆*w* according to the colour region. For example, for the blue colour, the sensitivity of the sensor increases eight times from load resistance *R*_L_ is 100 Ω to 510 Ω. As for the red colour, the sensitivity increases four times as load resistance *R*_L_ increases. Hence, the overall sensitivity in this study increases, as can be seen in 250 V, 500 V and 1000 V.

## 5. Conclusions

The OER acts as an element to present the mills or estates performance, where the overall process and of FFB needs to be considered especially during the reception and separation of the FFB. The current method implemented by the worker at the mills usually is by manual inspection using the eyes and visual inspection by the computer, which causes inconsistency, and it takes time to inspect the ripe FFB. Hence, proper technology or a sensor needs to be implemented during the screening of the FFB at the mills to identify the maturity of the oil palm fruit. This paper proposes a fruit battery sensor with a charging concept by applying and implementing different parameters to study the sensor sensitivity performance. On the basis the results, the specifications of the parameters such as load resistance *R*_L_, charging voltage *V*_c_ and charging time *t*_c_ tested give different data obtained from the experimental research. The difference voltage ∆*V*_avg_ is analysed when the moisture content difference ∆*W* is 50% to 80%. As the charging voltage *V*_c_ is tested from 250 V to 1000 V, the sensor performance sensitivity increases around 55%. Meanwhile, around 38% of sensitivity increases when the charging time *V*_c_ is tested from 5 s to 15 s. Furthermore, the performance of the sensor increases three-fold when 510 Ω is tested compared to 100 Ω. Thus, it can be concluded that the sensitivity of this fruit battery sensor is increased when the parameter conditions of load resistance *R*_L_, charging voltage *V*_c_ and charging time *t*_c_ are increased.

## Figures and Tables

**Table 1:** List of parameters tested.

Parameters	Values
Load resistance, *R*_L_	100 Ω
	510 Ω
Charging voltage, *V*_c_	250 V
	500 V
	1000 V
Charging time, *t*_c_	5 s
	10 s
	15 s
